# Researching the researchers: psychological distress and psychosocial stressors according to career stage in mental health researchers

**DOI:** 10.1186/s40359-022-00728-5

**Published:** 2022-02-01

**Authors:** Nicole T. M. Hill, Eleanor Bailey, Ruth Benson, Grace Cully, Olivia J. Kirtley, Rosemary Purcell, Simon Rice, Jo Robinson, Courtney C. Walton

**Affiliations:** 1 Telethon Kids Institute, 15 Hospital Avenue, Nedlands, WA Australia; 2 Centre for Child Health Research, The University of Western Australia, Perth, Australia; 3 School of Population and Global Health, University of Western Australia, Perth, Australia; 4 Orygen, Parkville, Melbourne, Australia; 5 Centre for Youth Mental Health, The University of Melbourne, Parkville, Australia; 6 School of Public Health, University College Cork, Cork, Ireland; 7 National Suicide Research Foundation, Cork, Ireland; 8 Department of Neuroscience, Center for Contextual Psychiatry, KU Leuven, Leuven, Belgium

**Keywords:** Mental health, Researchers, Wellbeing, University, Students, Stress

## Abstract

**Background:**

Although there are many benefits associated with working in academia, this career path often involves structural and organisational stressors that can be detrimental to wellbeing and increase susceptibility to psychological distress and mental ill health. This exploratory study examines experiences of work-related psychosocial stressors, psychological distress, and mental health diagnoses among mental health researchers.

**Methods:**

This international cross-sectional study involved 207 mental health researchers who were post-graduate students or employed in research institutes or university settings. Work-related psychosocial stressors were measured by the Copenhagen Psychosocial Questionnaire III (COPSOQ III). Psychological distress was assessed using the Depression-Anxiety-Stress Scale-21 (DASS-21). Thoughts of suicide was assessed using an adaptation of the Patient Health Questionnaire-9 (PHQ-9). History of mental health diagnoses was assessed through a custom questionnaire. Pearson’s chi-square test of independence was used to compare mental health diagnoses and suicidal ideation across career stages. The association between work-related psychosocial stressors and psychological distress was conducted using multivariate linear regression controlling for key demographic, employment-related and mental health factors.

**Results:**

Differences in ‘demands at work’ and the ‘work-life balance’ domain were lowest among support staff (*p* = 0.01). Overall, 13.4% of respondents met the threshold for severe psychological distress, which was significantly higher in students compared to participants from other career stages (*p* = 0.01). Among the subgroup of participants who responded to the question on mental health diagnoses and suicidal ideation (*n* = 152), 54% reported a life-time mental health diagnosis and 23.7% reported suicidal ideation since their academic career commencement. After controlling for key covariates, the association between the ‘interpersonal relations and leadership’ domain and psychological distress was attenuated by the mental health covariates included in model 3 (*β* = −0.23, *p* = 0.07). The association between the remaining work-related psychosocial stressors and psychological distress remained significant.

**Conclusions:**

Despite working in the same environment, research support staff report experiencing significantly less psychosocial stressors compared to postgraduate students, early-middle career researchers and senior researchers. Future research that targets key modifiable stressors associated with psychological distress including work organization and job content, and work-life balance could improve the overall mental health and wellbeing of mental health researchers.

## Introduction

The mental health and wellbeing of academic staff and students at higher education institutions (including universities) has become a prominent concern in the research community [1, 2]. Although there are many benefits and privileges associated with working in academia including knowledge gain, personal fulfilment, flexibility, and comparatively high salaries at senior levels, this career path often involves a range of structural and organisational stressors that may potentially compromise well-being and increase stress. Examples of academic-specific stressors identified in previous studies include being regularly evaluated and ‘benchmarked’ against output metrics, cyclical competition for funding (including salary support), job insecurity and uncertainty, and balancing multiple roles (teacher, mentor, researcher, writer, reviewer, manager, and administrator) [2–5]. These work commitments regularly interfere with personal life [5, 6] and have been shown in previous meta-analyses to be associated with increased psychological distress, and poor mental health outcomes such as depression and anxiety and suicidal ideation across occupations [7–9].

In response to increased international scrutiny of the academic work culture, several reports have been produced that highlight key work-place stressors experienced by researchers in academic settings [3, 10, 11]. For example, a report published by Wellcome Trust in the United Kingdom [3] highlighted concerns about career uncertainty and longevity, including a culture of long working hours, and continually changing goalposts with overwhelming and unrealistic expectations on productivity. Furthermore, a third of participants (34%) described accessing support from a mental health professional for depression or anxiety since working in academia [3]. This proportion was higher for females (38%) and non-binary respondents (66%), than males (25%). However, estimates of symptom prevalence and severity using validated clinical scales (e.g. DASS21) [12] were not collected nor was the association between symptom severity and work-related psychosocial stressors investigated.

To date, much of what is known about the mental health of academics stems from studies conducted among graduate student populations [13, 14]. However, it is often unclear what proportion of these samples conduct research, with many enrolled in applied study (e.g., medical training) that may not generalise to academics in research roles. Using the Generalized Anxiety Disorder Scale and Patient Health Questionnaire, Evans and colleagues [13] showed graduate research students were more than six times more likely to report experiencing symptoms of mental ill health including anxiety or depression than the general population, with rates of 39% and 41% respectively scoring in the ‘moderate’ or ‘severe’ range. Furthermore, psychosocial work-related factors such as poor work-life balance and poor mentoring relationships were revealed as being more common in those with a mental health disorder. Another study examined psychological distress [15] using the General Health Questionnaire, as well as job satisfaction, in a large sample of Australian university staff and found that 43% of academic staff scored above the cut-off [6], indicating increased risk of a possible mental health disorder [6, 16]. Together these findings suggest that the severity of psychological distress among academics, relative to the general population, may be related to modifiable work-related psychosocial stressors. Given psychological distress is characterised by depressive and anxiety symptoms, and is an indicator of mental ill health conditions such as depression and anxiety disorders [17] research that seeks to identify the association between work-related stresses and psychological distress may have upstream benefits that reduce the progression of a later mental health diagnosis.

Though there is now a growing concern about the mental health of researchers, particularly in early stages of their careers, the majority of work to date has focussed on assessment of stress, environmental factors, or relied on non-clinical instruments to measure researchers’ mental health [18]. Additionally, differences in psychological distress and mental health outcomes that are experienced at different career stages (e.g., postgraduate students versus senior researchers) has not been investigated. This gap in evidence is noteworthy since different modifiable work-related stressors may be more or less prevalent at different career stages. Given the link between job stress and the prospective development of psychological distress, mental ill health (e.g., depression and anxiety) and organisational productivity (e.g., sickness, absence rates, and workers compensation claims) [19], understanding the burden of psychological distress and mental ill health, including suicidal ideation, and work-related psychosocial stressors across different career stages has important implications for employees, as well as occupational health, safety regulators, insurers, unions and employers [20].

The current study was undertaken as part of the International Association for Suicide Prevention taskforce on emotional health and wellbeing. It is exploratory in nature, with the aim of investigating the work-related psychosocial stressors experienced by mental health researchers across different career stages, the prevalence of psychological distress and mental ill health, and the association between work-related psychosocial stressors and psychological distress within this population. In doing so, we seek to expand on the existing evidence-base in order to better identify the possible modifiable work-related psychosocial stressors that impact mental health researchers and identify possible opportunities for intervention and prevention of psychological distress and mental ill health among mental health researchers.

## Material and methods

### Data and sampling

This exploratory cross-sectional study used data from an online survey examining the association between work-related psychosocial stressors and psychological distress in an international sample of specific to mental health researchers. The study was approved by the University of Melbourne Human Research Ethics Committee (ID 1954670). All methods were performed in accordance with the relevant guidelines and regulations and all participants provided informed consent. The survey was available between 28 October 2019 and 1 March 2020. Participants were recruited via a number of targeted strategies including the email distribution lists of universities and mental health organisations, together with advertisements on the social media platform, Twitter. Recipients were invited to share the link to the study to their potentially eligible colleagues.

Eligible participants included those who were: (1) employed (full time, part time or casually) by a university or research institution (including research assistants, project managers, lecturers, and other academic staff), or; (2) enrolled as a postgraduate student (full-time or part-time Master’s or Ph.D. candidate), and; (3) the person’s main field of research related to mental health (e.g., psychology, psychiatry, social work). There were no restrictions on geographic location. All participants were screened against the eligibility criteria using an automatic skip-logic algorithm embedded within Qualtrics survey platform [21]. Participants who did not meet the eligibility criteria were not permitted to proceed to the study survey. The survey was formatted so that participants could not complete the survey more than once. Participation was anonymous and participants were not provided any incentive for taking part in the research.

A total of 357 participants provided consent, of whom 207 completed both the Copenhagen Psychosocial Questionnaire III (COPSOQ III) [22] and the Depression Anxiety and Stress Scale (DASS-21), representing 57.2% of the initial sample. Additional exploratory analyses were conducted on a subsample of 152 participants who answered questions about their history of mental health diagnoses.

## Measures

### Sociodemographic and work-related characteristics

Sociodemographic variables were assessed in a customised survey developed for the purpose of this study and included age, gender (male/female/other), relationship status (entered as a binary variable indicating the presence or absence of a relationship/spouse), dependents (e.g., children including biological and step-children; entered as binary variable representing the presence or absence of dependents), employment status (casual, full-time, part-time), type of work contract (fixed term/permanent) and clinical (e.g., registered psychologist or doctor) status (yes/no). Participants were classified according to their self-reported career stage. Participants who were employed as a research assistant or project officer were combined into a single category representing support staff. Participants who were enrolled in a Postgraduate degree (PhD or Master’s degree) were categorised as students. Participants who were employed at postdoctoral level or as a lecturer, were categorised as EMCRs. Lastly, senior researchers were participants who were employed as associate professor or above.

### Work-related psychosocial stressors

Work-related psychosocial exposures were assessed using the COPSOQ III [22]. The COPSOQ III was selected because it has been validated in over 14 countries worldwide [23] and assesses relevant dimensions (e.g., work-life balance) that have been identified as key work-related psychosocial stressors experienced by academics in research settings [3, 5]. Questionnaire items were obtained from the COPSOQ III middle and core items [24]. The questionnaire used in this study comprised 60 items, encompassing 25 psychosocial dimensions and five domains (Table 1) [22]. Each item is rated on a 5-point Likert scale. All items were transformed to a value ranging between zero (minimum value) to 100 (maximum value) with lower scores representing the lowest probable exposure (never/hardly ever) and 100 representing the highest probable exposure (always or to a very large extent). Higher scores indicated positive outcomes for the work organization and job content, interpersonal relations and leadership, social capital, and general health domains. Whereas higher scores indicated negative outcomes for the demands at work and work-life balance domains. Mean values were summarised according to the five core domains established previously in a previous international validation study that showed acceptable to good reliability with a Cronbach α > 0.7 [22] and good construct validity [25]. No adaption was made for this study.

**Table 1:** COPSOQ III Questionnaire domains and dimensions

Domains	Dimensions
Demands at work	Emotional demands
	Demands for hiding emotions
	Quantitative demands
	Work pace
Work organization and job content	Influence at work
	Possibilities for development
	Control over working time
	Meaning of work
Interpersonal relations and leadership	Recognition
	Predictability
	Role conflicts
	Role clarity
	Illegitimate tasks
	Quality of leadership
	Social support from supervisor
	Social support from colleagues
	Sense of community at work
Work individual interface (work-life balance)	Job insecurity
	Insecurity over working conditions
	Quality of work
	Job satisfaction
	Work-life conflict
Social capital	Horizontal trust
	Vertical trust
	Organizational justice
General health	Self-rated health

### General psychological distress

General psychological distress was assessed using the DASS-21 [12], a self-report measures of depression, anxiety and stress. The DASS-21 is an internationally validated instrument for measuring psychological distress [26] and has been shown as a valid and reliable tool for predicting the development of a possible mental health disorder in clinical settings [27]. Participants were asked to score each item on a 4-point Likert scale from 0 (did not apply to me at all) to 3 (applied to me very much). Total scores were computed by adding each item and multiplying the score by a factor 2 [12]. Total scores for the DASS-21 [12] range between zero and 120. Cut-off scores of 60 were labelled high distress [12]. Good inter-rater reliability, test–retest reliability, and validity of the DASS-21 have been reported previously in both clinical and non-clinical populations [28–30].

### Self-reported diagnosed psychological disorder

Self-reported history of diagnosed mental ill health was assessed using two questions developed specifically for this study: (1) Prior to beginning your research career (including your Ph.D.), have you ever been diagnosed with a psychological disorder? (2) Since beginning your research career (including your PhD), have you ever been diagnosed with a psychological disorder? Participants were provided with the response options ‘yes’, ‘no’, and ‘I have not been diagnosed, but I probably could have been’.

### Suicidal ideation

Self-reported suicidal ideation was assessed using three questions adapted from item 9 in the Patient Health Questionnaire-9 (PHQ-9; 31). Item 9 in the PHQ-9 [31] evaluates the frequency of suicidal ideation over the preceding two weeks and has been used as a single scale in studies reporting the prevalence of suicidal ideation [32, 33] and has shown to be a valid measure of suicidal ideation in studies comparting results with those from detailed clinical interviews [34–36]. In the present study, participants were asked: (1) Over the past two weeks, how often have you been bothered by thoughts that you would be better off dead, or thoughts of hurting yourself in some way? Response options were: Not at all, more than half the days, nearly every day and several days. Items were collapsed into a binary variable representing the presence (consisting of the responses: “more than half the days”, “nearly every day” and “several days” or absence (consisting of the response: “not at all”) of suicidal ideation for each item. Additionally, participants were asked: (2) Over the past year, have you experienced thoughts that you would be better off dead, or thoughts of hurting yourself in some way? and (3) Since beginning your research career (including during your Ph.D.), have you ever experienced thoughts that you would be better off dead, or thoughts of hurting yourself in some way? Participants responded ‘yes’ or ‘no’, indicating the presence or absence of suicidal ideation.

## Analysis

Descriptive analysis was conducted to determine the sociodemographic characteristics of the study participants and their history of mental health diagnoses, suicidal ideation, work-related psychosocial exposures and psychological distress. Pearson’s chi-square test of independence was used to compare mental health diagnoses and suicidal ideation across career stages (research support staff, postgraduate students, EMCRs, and senior researchers). Group comparisons of work-related psychosocial exposures, DASS-21 [12] psychological distress and related sub-scores were conducted using ANOVA. Multiple pairwise comparisons were performed using the Tukey post hoc test, stratified by career stage.

Multivariate linear regression models [37] were used to estimate the association between the five work-related psychosocial stressor domains and psychological distress, controlling for age, sex, career stage, employment type (fulltime, part-time, casual) and the presence of a mental health policy at work (yes, no, unsure) lifetime mental health-diagnoses (present, absent), suicidal ideation in the past two-weeks (present, absent) for the subsample of 152 participants with complete data. In model 1 the association between work-related psychosocial stressors and psychological distress was adjusted by age and sex (male, female). Model 2 was adjusted for age, sex career stage, hours of employment, employment type, and the presence of a mental health policy at work. Model 3 was adjusted for the covariates included in Model 1 and 2 as well as lifetime mental health diagnosis and suicidal ideation in the past 2 weeks. Coefficients of linear regression (*β*) are calculated and displayed along with their 95% confidence intervals. To identify differences across models we compared coefficients and confidence intervals to examine whether the models were statistically different. All analyses were conducted in R v 4.1.2.

## Results

### Sociodemographic and employment characteristics

Among the 357 participants who provided consent, 207 participants completed the full COPSOQ III [22). survey and DASS-21 [12]; a completion rate of 57.2%. Participants were from Australia (63.7%), Europe (29.9%), North America (5.3%), and South East Asia (< 1%). Most participants were female (82.1%) and over half (56.5%) were aged 18–34 years. Table 2 displays the sociodemographic and employment characteristics of participants according to career stage. The largest group of participants were postgraduate students (34.3%), followed by EMCRs (28.5%), senior researchers (20.3%), with research support staff constituting the smallest group (16.9%). One third (31%) of participants reported the presence of a mental health policy at their research institution, however relatively few had read the policy or were aware of its contents (15%).

**Table 2:** Sociodemographic and employment characteristics by career stage

	Total	Support staff	Student	EMCR	Senior researcher	Chi square
	N = 207	n = 35	n = 71	n = 59	n = 42	*p* value
		n (%)	n (%)	n (%)	n (%)	
*Gender*						
Male	35	7 (20.0%)	8 (11.3%)	7 (11.9%)	13 (31.0%)	0.05
Female	170	28 (80.0%)	61 (85.9%)	52 (88.1%)	29 (69.0%)	
Non-Binary	2	–	2 (2.8%)	–	–	
*Age range (years)*						
18–24	14	8 (22.9%)	8 (11.3%)	–	–	0.05
25–34	103	21 (60.0%)	51 (71.8%)	31 (52.5%)	–	
35–44	53	6 (17.1%)	7 (9.9%)	21 (35.6%)	17 (40.5%)	
45–54	24	–	3 (4.2%)	6 (10.2%)	15 (35.7%)	
55–64	12	–	2 (2.8%)	1 (1.7%)	9 (21.4%)	
65 +	1	–	–	–	1 (2.4%)	
*Relationship status*						
Cohabitating	52	4 (11.4%)	24 (33.8%)	19 (32.2%)	5 (11.9%)	0.05
Divorced	2	–	–	–	2 (4.8%)	
Married	78	6 (17.1%)	13 (18.3%)	31 (52.5%)	28 (66.7%)	
Registered partnership	4	2 (5.7%)	2 (2.8%)	–	–	
Separated	2	–	1 (1.4%)	–	1 (2.4%)	
Single	69	23 (65.7%)	31 (43.7%)	9 (15.3%)	6 (14.3%)	
*Dependents*						
Yes	57	4 (11.4%)	9 (12.7%)	16 (27.1%)	28 (66.7%)	0.00
No	150	31 (88.6%)	62 (87.3%)	43 (72.9%)	14 (33.3%)	
*Clinical degree*^a^						
Yes	45	3 (8.6%)	14 (19.7%)	15 (25.4%)	13 (31.0%)	0.10
No	157	32 (91.4%)	57 (80.3%)	45 (74.6%)	29 (79.0%)	
*Type of work contract*						
Permanent	46	3 (8.6%)	6 (8.5%)	16 (27.1%)	21 (50.0%)	0.00
Temporary/Fixed term	121	30 (88.6%)	30 (42.3%)	42 (71.2%)	19 (45.2%)	
Other	41	2 (5.7%)	36 (50.7%)	1 (1.7%)	2 (4.8%)	
*Employment status*						
Full time	156	26 (74.3%)	47 (66.2%)	48 (81.1	35 (83.3%)	0.48
Part time	38	7 (20.0%)	17 (23.9%)	9 (15.3%)	5 (11.9%)	
Other	12	2 (5.7%)	6 (8.5%)	2 (3.4%)	2 (4.8%)	
*Mental health policy present*						
Yes	64	16 (45.7%)	15 (30.5%)	18 (30.5%)	15 (35.7%)	0.06
No	100	11 (31.4%)	37 (52.5%)	31 (52.5%)	21 (50.0%)	
Unsure	43	8 (22.9%)	19 (17.0%)	10 (17.0%)	6 (14.3%)	
*Participant has read the mental health policy*^b^						
Yes	32	6 (37.5%)	7 (46.7%)	11 (61.1%)	8 (53.3%)	0.57
No	32	10 (62.5%)	8 (53.3%)	7 (38.9%)	7 (46.7%)	
*Thinks the mental health policy is adequate*^b^						
Yes	20	3 (18.8%)	6 (40.0%)	8 (44.4%)	3 (20.0%)	0.48
No	10	2 (12.4%)	3 (20.0%)	3 (16.7%)	2 (13.3%)	
Unsure	34	11 (68.8%)	6 (40.0%)	7 (22.9%)	10 (66.7%)	

*EMCR* Early-middle career researcher

^a^Includes participants who are currently completing a clinical degree (e.g., medicine, psychology or similar)

^b^Denominator is based on the number of participants who were aware of their organisation having a mental health policy

### Work-related psychosocial exposures

Table 3 shows the work-related psychosocial exposures according to career stage for the five work-related COPSOQ III [22] domains (see Table 1). Differences between career stages were observed for the domains: ‘demands at work’, ‘work-life balance (termed hereafter as work-life balance) ‘social capital’ and ‘health and wellbeing.’ Tukey’s post-hoc analysis revealed that the differences in the ‘demands at work domain’ were driven by lower (i.e., better) scores among research support staff relative to other career stages (*p* < 0.001 for postgraduate students, EMCRs and senior researchers, respectively). A similar trend was observed for the ‘work-life balance’ domain (*p* < 0.001 for postgraduate students; *p* = 0.002 for EMCRs and *p* = 0.04 for senior researchers). Differences in social capital were driven by higher scores among research support staff compared to senior researchers (*p* = 0.004). Lastly, differences in health and wellbeing were driven by higher scores in research support staff compared to postgraduate students (*p* = 0.003) and in senior researchers compared to students (*p* = 0.03).

**Table 3:** COPSOQ III Work-related psychosocial exposures by career stage

	Support staff		Student		EMCR		Senior researcher		ANOVA
	Mean	SD	Mean	SD	Mean	SD	Mean	SD	*p* value
Demands at work	41.55	16.54	53.00	17.38	52.82	13.92	54.32	9.77	0.006
Work organization and job content	71.65	12.75	73.89	16.70	73.89	11.86	75.18	9.36	0.719
Interpersonal relations and leadership	68.13	11.49	64.51	16.37	62.92	13.04	62.73	12.93	0.270
Work-life balance	40.00	11.14	49.87	11.07	48.23	10.78	48.43	10.44	< 0.001
Social capital	66.19	16.22	57.75	23.08	56.14	16.49	51.19	17.46	0.008
Health and wellbeing	70.14	23.01	51.74	27.94	57.63	21.90	64.88	22.13	< 0.001

*EMCR* Early-middle career researcher

### General psychological distress

**Fig. 1:**
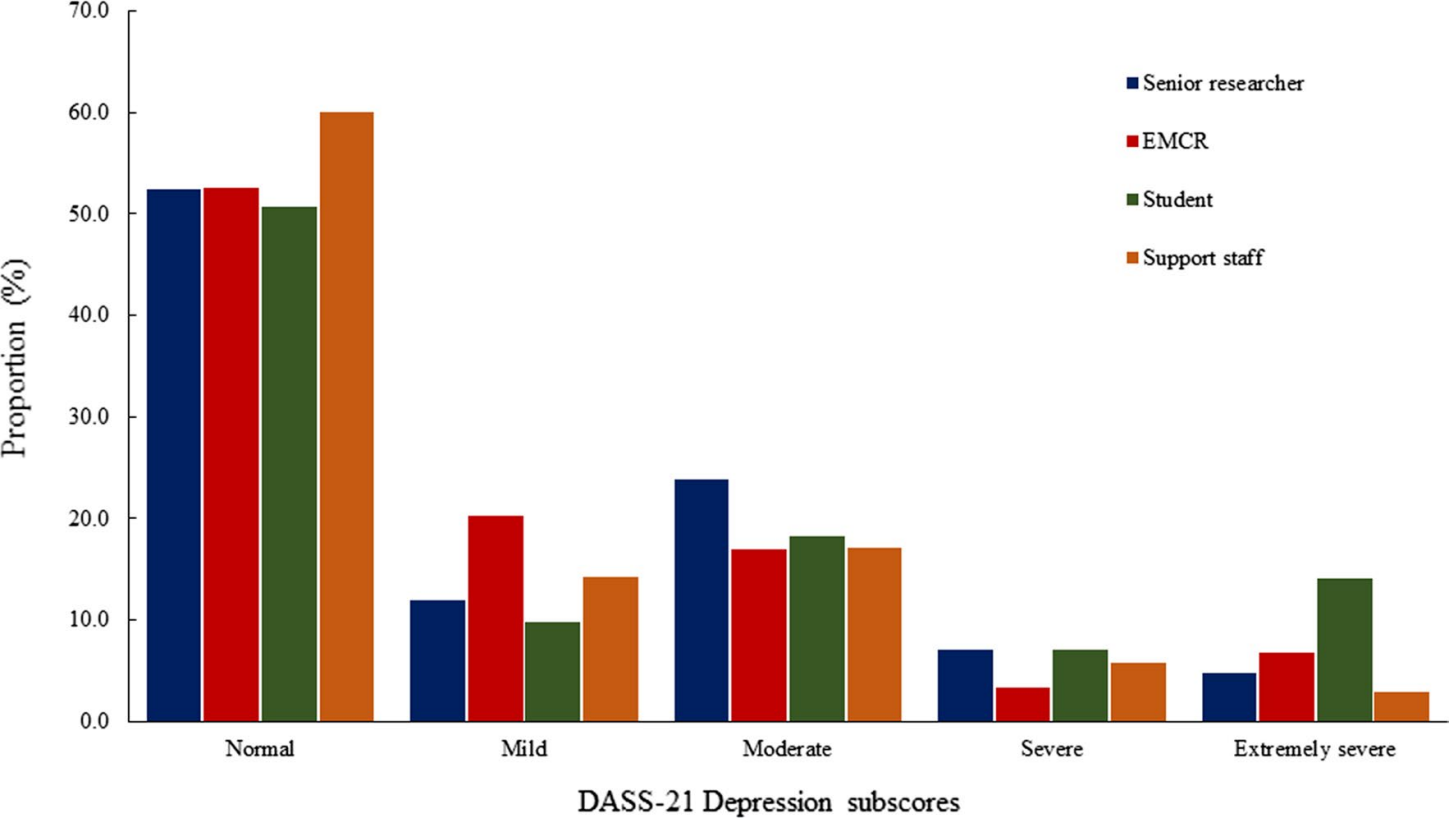
DASS-21 depression subscores by career stage

**Fig. 2:**
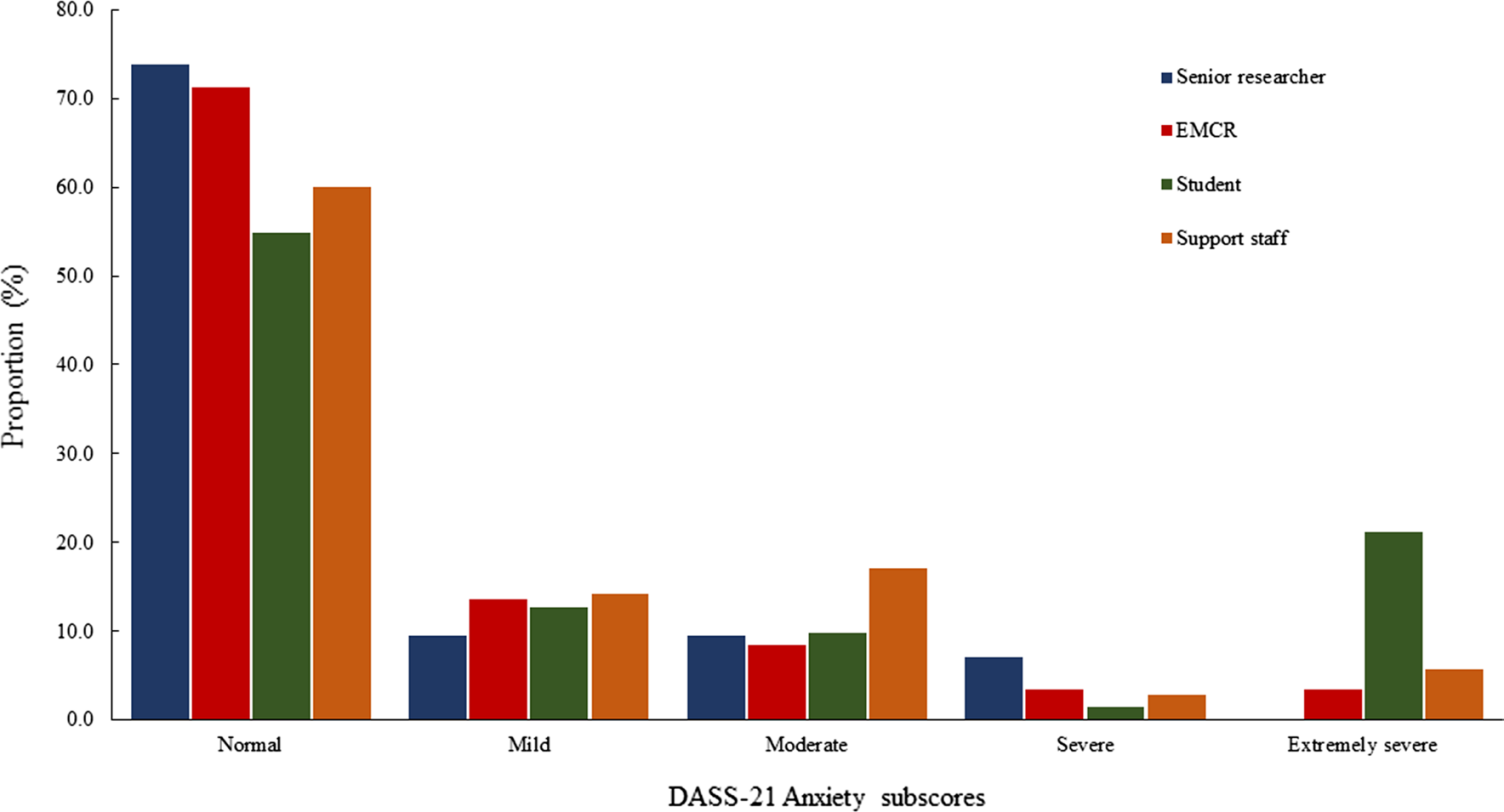
DASS-21 anxiety subscores by career stage

**Fig. 3:**
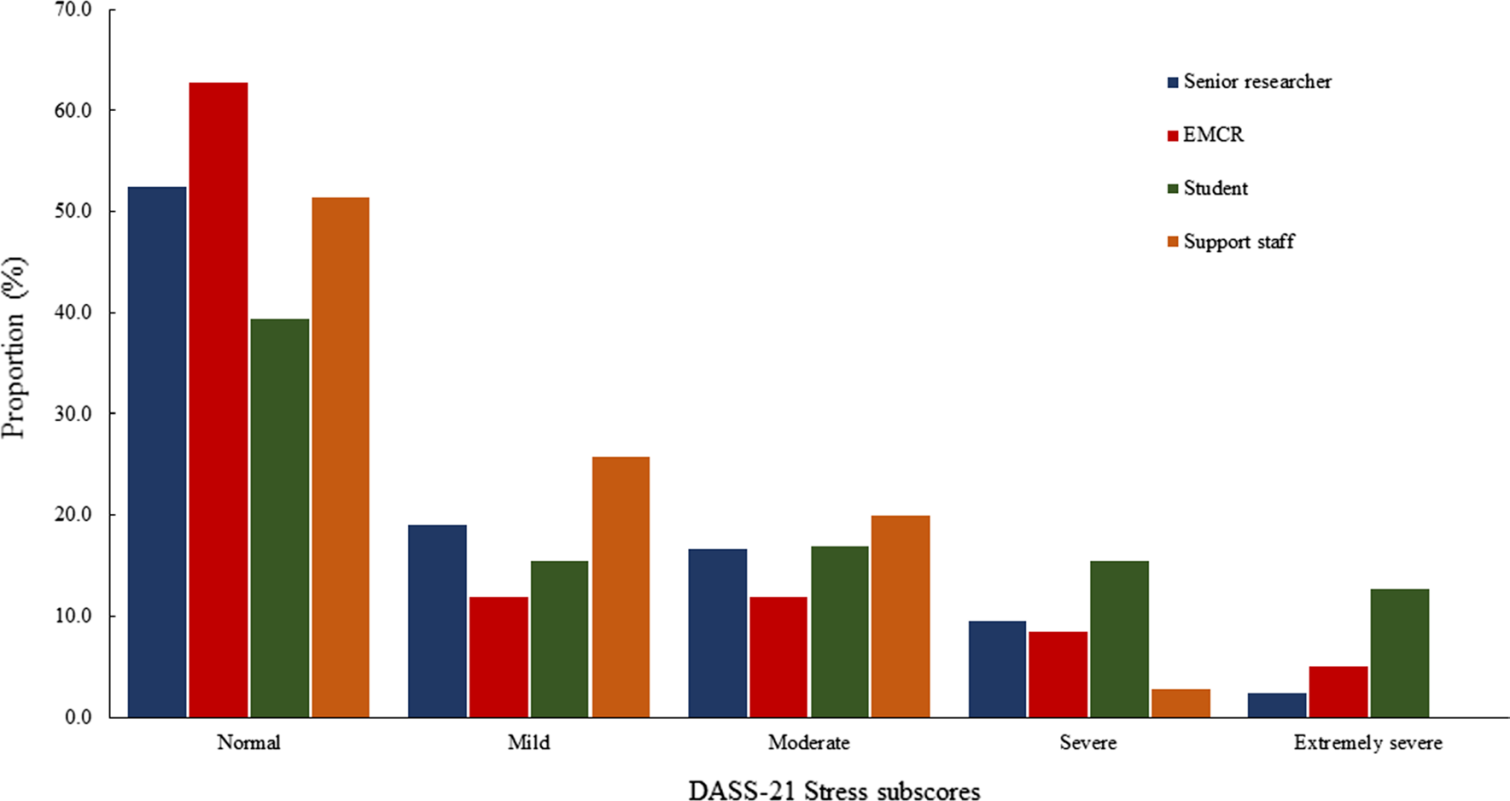
DASS-21 stress subscores by career stage

Figures 1, 2 and 3 show sub-scores for depression, anxiety, stress, and total psychological distress measured by the DASS-21 [12], stratified by career stage. Post-hoc comparisons revealed postgraduate students reported experiencing significantly greater anxiety and stress, and total psychological distress compared to research support staff (*p* = 0.01), EMCRs (*p* = 0.01) and senior researchers (*p* = 0.01; Table 4). A total of 27 (13.4%) participants reported DASS-21 [12] scores ≥ 60, indicating severe distress. Severe distress was most frequently reported among postgraduate students (*n* = 16), followed by research support staff (*n* = 4), EMCRs (*n* = 3) and senior researchers (*n* = 4). Fisher’s exact test revealed these differences were statistically significant (*p* = 0.02).

**Table 4:** DASS-21 scores stratified by career stage

	Support staff		Student		EMCR		Senior researcher		ANOVA
	Mean	SD	Mean	SD	Mean	SD	Mean	SD	*p* value
Depression	8.80	7.70	12.72	11.98	9.32	8.75	10.00	8.75	0.128
Anxiety	7.03	6.35	10.37	11.15	5.49	6.34	4.62	5.35	< 0.001
Stress	12.91	7.90	19.35	10.22	13.49	9.49	14.43	8.39	< 0.001
Psychological Distress	28.74	18.46	42.48	29.75	28.31	20.13	29.05	18.21	0.001

*EMCR* Early-middle career researcher

### Self-reported history of mental health diagnoses and suicidal ideation

Of the 152 participants who responded to the question on self-reported mental health diagnoses, over half (54.6%) had received a mental health diagnosis at some point during their lives and a further 46 (30.1%) reported a suspected mental health disorder (i.e., did not receive a diagnosis but thought they should have; Table 5). The proportion of participants who had a diagnosed mental health disorder prior to their academic career was over one-third (37.5%), while just under one-third (31.6%) of participants received a psychological diagnosis since commencing their academic career. Senior researchers were significantly less likely to report having received a mental health diagnoses prior to their career in academia, compared to research support staff, postgraduate students, and EMCRs. Of the 80 (52.0%) participants who reported suicidal ideation since embarking on their academic career, 36 (17.4%) reported experiencing suicidal ideation in the past fortnight and 69 (33.3%) reported experiencing suicidal ideation in the past year. All measures of suicidal ideation were comparable across career stages.

**Table 5:** Mental health history and suicidal ideation by career stage

	Total	Support staff	Student	EMCR	Senior researcher	Chi square
	N = 152	(n = 26)	(n = 57)	(n = 40)	(n = 29)	*p* value
	n (%)	n (%)	n (%)	n (%)	n (%)	
Diagnosed mental health disorder (lifetime)	83 (54.6%)	16 (61.5%)	33 (57.9%)	23 (57.5%)	11 (37.9%)	0.138
Suspected mental health disorder (lifetime)	46 (30.3%)	6 (23.1%)	15 (26.3%)	15 (37.5%)	10 (34.5%)	
No diagnosed or suspected mental health disorder (lifetime)	23 (15.1%)	4 (15.4%)	9 (15.8%)	2 (5.0%)	8 (27.6%)	
Diagnosed mental health disorder prior to academic career	57 (37.5%)	14 (53.8%)	26 (45.6%)	14 (35.0%)	3 (10.3%)	0.008
Suspected mental health disorder prior to academic career	48 (31.6%)	6 (23.1%)	13 (22.8%)	17 (42.5%)	12 (41.4%)	
No diagnosed or suspected mental health disorder prior to academic career	47 (31.0%)	6 (23.1%)	18 (31.6%)	9 (22.5%)	14 (48.3%)	
Diagnosed mental health disorder since beginning academic career	48 (31.6%)^a^	7 (26.9%)^b^	16 (28.1%)^c^	16 (40.0%)^d^	9 (31.0%)^e^	0.478
Suspected mental health disorder since beginning academic career	50 (32.9%)	8 (30.8%)	18 (31.6%)	16 (40.0%)	8 (27.6%)	
No diagnosed or suspected mental health disorder since beginning academic career	54 (35.58%)	11 (32.3%)	23 (40.3%)	8 (20.0%)	12 (41.4%)	
Suicidal ideation (past 2-weeks)	36 (23.7%)	4 (15.4%)	16 (28.1%)	8 (20%)	8 (27.6%)	0.519
Suicidal ideation (past 12-months)	69 (45.4%)	13 (50%)	26 (45.6%)	15 (37.5%)	15 (51.7%)	0.631

*EMCR* Early-middle career researcher

^a^n = 22 participants who reported mental health diagnoses both prior and since commencing their academic career

^b^n = 5 participants who reported MH diagnoses both prior and since commencing their academic career

^c^n = 9 participants who reported MH diagnoses both prior and since commencing their academic career

^d^n = 7 participants who reported MH diagnoses prior and since commencing their academic career

^e^n = 1 participant who reported MH diagnoses prior and since commencing their academic career

### Association between work-related psychosocial stressors and psychological distress

Table 6 shows the results of the regression models examining the relationship between work-related psychosocial exposures and psychological distress. A comparison of the confidence intervals for each of the models included in the analysis did not reveal statistically meaningful differences. After adjusting for all covariates, the association between ‘interpersonal relations and leadership’ and psychological distress was attenuated by the mental health covariates included in model 3 (*β* = −0.23, *p* = 0.07). The association between the remaining work-related psychosocial domains and psychological distress remained significant. Based on the standardised *β* coefficients from the fully adjusted model (model 3), the strongest associations were observed for ‘work organisation and job content’ (*β* = −0.27, *p* < 0.001) and ‘work-life balance’ (*β* = 0.23, *p* = 0.01) domains. The weakest association was observed in the social capital dimension (*β* = −0.10, *p* = 0.03).

**Table 6:** Multivariate linear regression estimates the COPSOQ III domains and DASS-21 psychological distress outcome

	Model 1			Model 2			Model 3		
	*β* coefficient	95% CI	*p* value	*β* coefficient	95% CI	*p* value	*β* coefficient	95% CI	*p* value
Demands at work	0.192		< 0.001	0.155		0.020	0.135		0.030
Work organization and job content	− 0.274	− 0.41 to − 0.15	0.001	− 0.275	− 0.41 to − 0.14	< 0.001	0.275	− 0.36 to − 0.11	< 0.001
Interpersonal relations and leadership	− 0.156	− 0.28 to − 0.03	0.020	− 0.148	− 0.28 to − 0.02	0.020	− 0.114	− 0.24 to 0.002	0.070
Work-life balance	0.310	0.16 to − 0.47	< 0.001	0.272	0.11 to 0.44	0.001	− 0.229	0.07 to 0.39	0.010
Social capital	− 0.141	− 0.23 to − 0.05	0.003	− 0.012	− 0.21 to − 0.03	0.010	− 0.101	− 0.19 to − 0.01	0.030
Health and wellbeing	− 0.204	− 0.27 to − 0.13	< 0.001	− 0.194	− 0.26 to − 0.13	< 0.001	− 0.169	− 0.23 to − 0.10	< 0.001

Model 1 was adjusted for age and sex. Model 2 was adjusted for career stage, hours of employment, employment type, and the presence of a mental health policy at work. Model 3 was adjusted for lifetime mental health diagnosis and suicidal ideation in the past 2 weeks

*CI* Confidence interval

## Post-hoc power analysis

The post-hoc power analysis revealed that with 4 groups, a medium effect size 0.3, and a power of 0.8, the recommended sample size for the ANOVA was 44 for each group. The estimated power for the regression analysis was 0.9, based on 152 participants, 8 covariates, and a medium effect size of 0.3.

## Discussion

This study sought to describe the psychological distress, mental health and work-related psychosocial stressors experienced by mental health researchers according to their career stage and to identify the association between general psychological distress and work-related psychosocial stressors within the academic settings. Results of the regression analysis provide some insight into the potential modifiable work-related stressors associated with psychological distress among mental health researchers. For example, the strongest associations between psychological distress and work-related psychosocial stressors occurred in the ‘work organization and job contents’ and ‘work-life balance’ domains. The ‘work organization and job contents’ domain include factors such as influence at work, possibilities for development and control over working time, whereas the ‘work-life balance’ domain comprises commitment to the workplace, work engagement, job insecurity, insecurity over working conditions (e.g., office and desk space availability), quality of work, job satisfaction, and work-life-conflict. The current findings corroborate and extend on those reported in a previous survey involving 4,267 researchers in the UK that showed long-working hours, competing demands which reduce capacity to conduct research, and lack of job security as key concerns faced by academics [3]. Our study extends these findings by showing that after controlling for demographic, employment, and mental health factors, the same work-related psychosocial stressors are associated with increased psychological distress.

Results of the descriptive analysis of mental health and suicidal ideation outcomes revealed that over half of participants had either received a mental health diagnosis in their lifetime or had a suspected mental health diagnosis, compared to approximately 18% to 36% reported in previous studies in the general population [38, 39]. Moreover one-third of participants had received a mental health diagnosis since commencing their academic careers. Similarly, rates of suicidal ideation were reported among 52% of participants, compared to approximately 10% reported in a previous cross-sectional study of suicidal ideation in the general population [40]. Taken together, these findings suggest that many mental health researchers have lived experience of mental ill health themselves, and that the work-place environment remains an important setting for primary and secondary prevention of mental-ill health.

This study showed that rates of self-reported mental health diagnoses and suicidal ideation were comparable across career stages for those in employment and the post-hoc power analysis demonstrated that these findings are unlikely to be driven by power limitations. However, postgraduate students reported notably higher scores for psychological distress, as well as anxiety, depression, and stress sub-scores, compared to research support staff, EMCRs and senior researchers. Potential explanations include financial strains experienced by many postgraduate students, which may include the need to also engage in paid employment leading to multiple role commitments [41]. Another possibility is that postgraduate students may face greater uncertainty regarding future employment [14, 41]. Indeed, previous studies have shown that although the number of PhD graduates from science, technology engineering and mathematics has increased substantially over the past 20-years [42], the number of post-graduate research positions has remained constant, resulting in fewer job prospects among recent graduates [43]. Due to missing data on mental health outcomes it was not feasible to investigate the association between work-related psychosocial stressors and self-reported mental health diagnoses. However previous meta-analytic evidence across occupation groups found factors such as effort-reward imbalance and job insecurity were associated with a 1.81 and 1.91 increased odds of suicidal ideation [8], whereas factors such as long working hours and job insecurity were associated with 1.31–1.77 increased odds of developing an anxiety disorder [8].

It is noteworthy that senior researchers in this study were also significantly less likely to have received a mental health diagnosis prior to their career in academia compared to postgraduate students and EMCRs. On the one hand, it is possible that mental health researchers who stay in academia and transition to senior roles with tenure are those who are less likely to face ongoing work-related stressors that may contribute to their risk of psychological distress or mental ill health [44]. It is also possible that students and EMCRs experience significant differences in career pressure and funding success decline that senior researchers did not experience, to the same extent [45].

It has been argued that key structural changes within University institutions such as the marketisation of university education; increased competition between institutions; changes to higher education consumption patterns; the commodification of education; and the growth of managerialism is associated with negative work culture and reduced mental health and wellbeing in recent decades [5]. These structural changes have corresponded with increased student numbers, more demanding students, increased teaching demands, and a shift towards metrics-based performance management [5]. Similar findings were reported in the recent Wellcome trust report into academic work-place culture which identified the tendency for risk aversion and short termism among research institutions, manifested by short term contracts, job insecurity, increased competition to secure limited funding as significant concerns among academic researchers [3]. Moving forward, it is imperative that academic institutions reflect on the impact that structural barriers have on the workplace culture among academics and invest in strategies that have the potential to mitigate the adverse effects associated with psychological distress and wellbeing.

Despite the current recommendations, changes to the institutional structures require time and strategic investment, both of which are unlikely to occur rapidly. Thus it is important that the sector consider interventions that can be implemented in the interim, to bridge the gap between existing work-related psychosocial stressors and wellbeing among academics. Whilst evidence regarding the effectiveness of interventions targeting mental ill health in the workforce is limited, previous studies have shown that screening employees for mental ill health symptoms, proactive outreach, and providing opportunities for therapeutic counselling in the workplace, is both cost effective and associated with improved individual mental health outcomes and workplace productivity [46, 47]. Furthermore, secondary interventions such as stress management, coping, resilience training, mindfulness-based stress reduction, problem solving, physical activity and cognitive behavioural therapy have been efficacious at increasing productivity and reducing distress in other occupational settings [48–52]. Given less than half of participants in the current study indicated having knowledge of a mental health and wellbeing policy or strategy at their place of employment, an important next-step forward for research institutions is to assess for the presence or absence of mental health and wellbeing policies within the workplace. This includes ensuring that mental health researchers have both access to and knowledge of help-seeking pathways at their institution or place of employment [53] and having policies in place that facilitate employees return to work following an episode of mental ill health [54]. Importantly these policies should include proactive strategies to reduce stigmatizing attitudes and cultures of non-disclosure that have been shown to impact individuals help-seeking behaviours in the workplace [55].

Lastly, data reported in the present study were collected prior to the onset of the COVID-19 pandemic. Factors such as social-distancing restrictions and the transition from office-based to home-based work environments have been linked to disruptions in productivity across disciplines [56]. As such, it is likely that the psychosocial stressors experienced by mental health researchers, such as those involving the work-life balance have increased as a result of COVID-19 restrictions. These effects may be particularly pronounced among specific groups, such as academics with young dependents [57, 58] as well as postgraduate students who may have experienced significant disruptions in their social support networks whilst working remotely during their studies. Moving forward, future research that examines the impact of the COVID-19 pandemic on the mental health of mental health researchers and academics, more generally, should be prioritised so that decision makers within research institutions can embed timely and appropriate primary and secondary harm minimization strategies, accordingly.

### Limitations

Limitations exist within this study. First, the majority of the sample were from western countries including Australia, UK and USA, with less than 1% from South East Asia and surrounding geographies. Significant cultural differences may exist in geographic regions not captured by the present survey and remain an important consideration for future studies. Second, the present study was limited to the 57% of participants who had completed the COPSOQ III [22] questionnaire and selection bias arising from missing data, particularly on suicide ideation outcomes, meant that it was not possible to investigate the association between mental health outcomes and work-related psychosocial stressors such as job insecurity and suicidal ideation, which have been reported in previous workplace studies [59]. Because attrition was greater than 40% it was not considered methodologically valid to use statistical adjustments such as multiple imputation on missing data [60]. For this reason the results of the present study should be interpreted in the context of generating hypotheses for future research [60].

Third, participants included in this study were self-selected and did not represent a random sample, nor did we sample participants for maximum variation. Furthermore, since participants were recruited via multiple email distribution links and via social media, it was not possible to identify the number of people who were contacted or reached, nor was it possible to calculate rates of refusal. This limitation is means that the study findings may be prone to selection bias and should be interpreted accordingly.

Lastly, previous studies have shown that occupation-based surveys may be susceptible to response biases reflecting higher rates of psychological distress compared to outcomes reported in population-based surveys [61]. This is considered to be a reflection of employees being consciously or unconsciously more inclined to vent their frustrations at their current work [61]. However, Winefield, Gillespie [6] found evidence to suggest that respondents to a university-based survey on stress and psychological distress were neither more nor less likely to display bias in their response based on their current distress. Given the current sample comprises mental health researchers who, by virtue of the academic and mental health training, may be more aware of response biases compared to the general population, we do not expect the results on general psychological distress to be significantly impacted by individual response biases. Nonetheless, as with any self-reported outcomes, results of the present study should be interpreted with caution.

### Conclusion

Over half of mental health researchers have experienced mental ill health during their lives and this figure is greater than those reported in the general population and this warrants concerted efforts to validate these findings against larger, representative samples within academia. Despite working in the same environment, research support staff experience significantly less psychosocial stressors compared to postgraduate students, early-middle career researchers and senior researchers. In contrast, students are significantly more likely to experience mental ill health and suicidal ideation relative to mental health researchers at different career stages. Future research that targets the modifiable stressors at each career stage, including key systemic issues linked to work organization and job content and those that impact work-life balance has the potential to improve the overall mental health and wellbeing of mental health researchers and that these differences ought to be reflected in mental health and wellbeing policy and practice within research institutions.

## Data Availability

The dataset used and analysed during the current study available from the corresponding author on reasonable request.
